# Clinical Considerations in Orthodontically Forced Eruption for Restorative Purposes

**DOI:** 10.3390/jcm10245950

**Published:** 2021-12-18

**Authors:** Grace Huang, Min Yang, Mohammad Qali, Tun-Jan Wang, Chenshuang Li, Yu-Cheng Chang

**Affiliations:** 1 Department of Orthodontics, Harvard School of Dental Medicine, Boston, MA 02115, USA; gracehuang@hsdm.harvard.edu; 2 Department of Periodontics, School of Dental Medicine, University of Pennsylvania, Philadelphia, PA 19104, USA; my851@upenn.edu (M.Y.); qali@upenn.edu (M.Q.); 3 Department of Orthodontics, School of Dental Medicine, University of Pennsylvania, Philadelphia, PA 19104, USA; tjwang@upenn.edu

**Keywords:** orthodontic extrusion, restoration, crown lengthening, forced eruption, implant site development

## Abstract

For restorations on teeth involving invasion of the supracrestal tissue attachment (biological width), as well as for lack of ferrule effect, crown lengthening is required for long-term periodontal health and success of the restoration. In the same fashion, site development is often necessary prior to implant placement in order to provide optimal peri-implant soft and hard tissue architecture conducive to future esthetics and function. Orthodontic extrusion, also known as forced eruption, has been developed and employed clinically to serve the purposes of increasing the clinical crown length, correcting the periodontal defect, and developing the implant site. In order to provide comprehensive guidance on the clinical usage of this technique and maximize the outcome for patients who receive the dental restoration, the currently available literatures were summarized and discussed in the current review. Compared to traditional crown lengthening surgery, forced eruption holds advantages of preserving supporting bone, providing improved esthetics, limiting the involvement of adjacent teeth, and decreasing the negative impact on crown-to-root ratio compared to the traditional resective approach. As a non-invasive and natural technique capable of increasing the available volume of bone and soft tissue, forced eruption is also an attractive and promising option for implant site development. Both fixed and removable appliances can be used to achieve the desired extrusion, but patient compliance is a primary limiting factor for the utilization of removable appliances. In summary, forced eruption is a valuable treatment adjunct for patients requiring crown lengthening or implant restorations. Nonetheless, comprehensive evaluation and treatment planning are required for appropriate case selection based upon the known indications and contraindications for each purpose; major contraindications include inflammation, ankylosis, hypercementosis, vertical root fracture, and root proximity. Further studies are necessary to elucidate the long-term stability of orthodontically extruded teeth and the supporting bone and soft tissue that followed them.

## 1. Introduction

Crowded, irregular, and projecting teeth have plagued people for millennia; indeed, attempts to correct these anomalies trace back to at least 1000 BC, in ancient Egypt [1]. Today, malocclusion, defined as “irregularity concerning teeth alignment and/or their relationship during dental occlusion beyond the range of what is accepted as normal,” affects more than half of the world’s population [2]. Since its professional inception in 1900 as the first dental specialty, founded by Dr. Edward H. Angle, orthodontics has become one of the most commonly sought-after dental procedures.

While a variety of orthodontic tooth movements are possible, it is imperative to identify the treatment purpose and the types of movements necessary to achieve the desired goals. Orthodontic extrusion, also known as forced eruption, is defined as alteration of tooth position by applying tractional forces in all regions of the periodontal ligament to stimulate marginal apposition of crestal bone [3]. The theory first originated in 1940, when Oppenheim observed the crestal apposition of bone in an extruded tooth of a young patient, and reported that the bone followed the occlusal movement of the tooth, thereby increasing the height of the alveolar crest [4]. In 1973, intentional forced eruption was proposed by Heithersay initially as a method of managing transverse root fractures using combined endodontic-orthodontic treatment [5]. That same year, Brown applied orthodontic treatment for periodontal purposes, and described the ability of the attachment apparatus to remodel under controlled forces, correct infra-bony defects, and achieve pocket reduction [6]. Around the same time, Ingber published articles discussing the application of forced eruption in treating non-restorable teeth as a result of trauma, caries or iatrogenic dentistry [7].

From the standpoint of lasting periodontal health, there are several situations in which the restoration of a compromised tooth may require crown lengthening; these usually involve the restorability of the tooth and the supracrestal tissue attachment (biologic width). If caries excavation has reached subgingivally as to invade the supracrestal tissue attachment biologic width, even a restoration with perfect marginal adaptation may still result in chronic gingival inflammation and bone loss [8,9,10]. The same is true in cases of horizontal fractures, internal and external resorptions, or perforations that extend into the biologic width. For full coverage restorations, the need for an adequate ferrule entails additional consideration [8].

The prevailing option for increasing clinical crown height has been osseous surgery, in which the supporting bone around the tooth is resected. While this procedure predictably lengthens the clinical crown of the tooth to be restored, it does so at the expense of the adjacent teeth—to create favorable bone architecture or positive architecture, the bone must be removed not only around the tooth in question but from the teeth on either side as well [11]. As a consequence, soft tissue recession may arise, and the patient may complain of sensitivity from the additional gingival recession [6]. In 1974, Ingber introduced forced eruption as a less invasive alternative for management of these otherwise non-restorable teeth that could avoid jeopardizing adjacent teeth and anatomy [7]. This technique for crown lengthening was further improved by Pontoriero in 1987 with the description of supracrestal fiberotomy to prevent concomitant migration of the periodontium of the extruded tooth [12].

Less than two decades later, in 1993, Salama and Salama proposed a systematic approach for applying forced eruption in another area [13]. In the interest of providing optimal peri-implant soft and hard tissue architecture for long-term implant health and favorable esthetics, site development is required in many instances. Surgical procedures such as guided bone regeneration, onlay grafts, and sinus augmentation have been reported to produce predictable results for horizontal ridge augmentation, but vertical ridge augmentation has proven more difficult [14]. Although distraction osteogenesis has seen more success in increasing bone height comparing to vertical ridge augmentation, its complexity and invasiveness have prevented it from being widely used [15]. Orthodontic extrusion/forced eruption is, therefore, an attractive option in cases where an implant will be replacing an existing, compromised tooth. In comparison to the aforementioned procedures, it is a less invasive and natural technique for increasing available soft and hard tissue volume [15,16,17].

Although many clinicians acknowledge the importance of clinical crown height in the long-term success of dental restorations and in the amount of available hard and soft tissue for future implant placement, forced eruption is not a commonly employed procedure. This may be due to the lack of evidence-based guidelines for case selection and management and the limited knowledge of possible complications. The goal of this review was to consolidate the clinical recommendations and contraindications for the use of forced eruption as a treatment option adjunct for crown-lengthening and implant site development.

## 2. Indications

### 2.1. Ferrule/Crown Lengthening

#### 2.1.1. History

The dental ferrule concept was first proposed by Rosen in 1961 as a means of prevention against “shattering of the root” [18]. This circumferential collar of gold that extends apical to the gingival seat of any core build-up material was hypothesized to reinforce endodontically treated teeth [18]. In 1990, Sorensen and Engelman discussed the ability of a ferrule to resist functional lever forces, wedging effects of tapered posts, and lateral stresses during post insertion [19]. It is generally accepted today that 1.5–2 mm of ferrule and 4.5 mm of supra-alveolar tooth structure are required for long-term restorative success; the difference is accounted for by approximately 2 mm of biological width [10,19]. When these requirements are not met, the procedure of crown lengthening surgery and orthodontically forced eruption are frequently performed. While crown lengthening has been the more popularly practiced option due to the simplicity and efficiency, many clinicians have demonstrated success with forced eruption since it was first utilized by Ingber in 1974 [7,20,21,22,23].

#### 2.1.2. Advantages and Disadvantages

**Figure 1:**
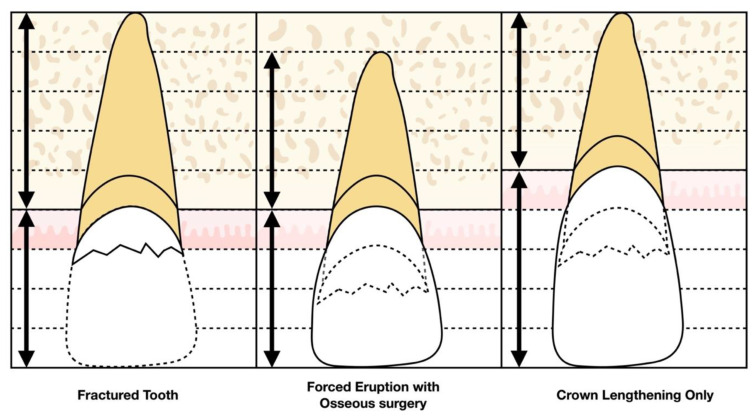
Comparison of crown-and-root ratio between crown lengthening only (5:4) and extrusion with osseous surgery or fiberotomy (4:4) when treating a fractured tooth with inadequate ferrule for full coverage restoration.

In comparison to crown lengthening surgery, forced eruption limits the involvement of adjacent teeth, preserves supporting bone, and avoids the potential complication of recession secondary to surgery. Furthermore, it does not negatively impact crown-to-root ratio as much as the resective approach [15,16,21,22] (Table 1). For example, if a tooth has a ratio of 4:5 preoperatively, and one unit of bone is removed to establish ferrule, then the postoperative ratio will be 5:4, which is unfavorable. In contrast, if the tooth is extruded the same unit and fiberotomy or post-extrusion crown lengthening is completed, the postoperative crown-to-root ratio will be 4:4 after accounting for occlusal reduction, which is more acceptable (Figure 1). By avoiding unexpected recession on the adjacent teeth and improving the crown-to-root ratio, the final esthetics of a tooth treated by forced eruption are superior to those of a tooth treated by crown lengthening surgery.

These pronounced advantages are counterbalanced by the prolonged treatment duration and the potential need for endodontic treatment in cases involving significant extrusion and subsequent occlusal reduction. Moreover, while repeat supracrestal fiberotomy reduces the need for crown lengthening surgery on the extruded tooth, it may not prevent coronal migration of the attachment apparatus altogether [15,16,21,22]. Indeed, crown lengthening surgery may still be indicated on the tooth upon completion of the extrusion [12,20]. It is noteworthy, however, that this surgery does not jeopardize the periodontium of the adjacent teeth to the extent that osseous surgery without prior forced eruption does (Table 1).

#### 2.1.3. Indications and Contraindications

With individualized and comprehensive treatment planning, orthodontically forced eruption can be an extremely valuable adjunct. Indications for orthodontic extrusion for crown lengthening or ferrule effect include subgingival or intraosseous carious lesions; isolated periodontal defects; and restoration of teeth with caries, horizontal fractures, perforations, or internal and external root resorptions that would violate biologic width [15,16]. Forced eruption can increase accessibility to these lesions, reduce periodontal defects, and facilitate tissue health around the final restorative material [15,16,21,22,24].

As always, orthodontics should not be initiated on a tooth with active periodontal inflammation, which may worsen tooth stability along with the treatment. Additional contraindications include ankylosis and hypercementosis, which would instead lead to intrusion of the adjacent anchor teeth [25]. Vertical root fracture typically indicates a hopeless prognosis requiring extraction of the tooth, and is therefore a contraindication for forced eruption in most of the cases [3,21]. Anatomy plays a key role in case selection as well, as root proximity and potential furcation exposure are relative contraindications. Moreover, candidate teeth should be evaluated for crown-to-root ratio and interocclusal space; extremely short roots that would not allow for adequate support of a final restoration and insufficient prosthetic space would likewise contraindicate extrusion [3] (Table 2).

### 2.2. Implant Site Development

#### 2.2.1. History

Although Dr. Branemark is credited with placing the first iteration of the modern endosteal implant in 1965, humans have in fact been toying with the idea of dental implants for millennia; there is evidence of Mayans embedding shells in the mandible around 600 AD, and Hondurans using stones to replace mandibular teeth around 800 AD. The history of dental implants also encompasses the development of eposteal and transosteal implants beginning around 1940. Indeed, the modern dental implants now being placed in excess of 5 million per year are the result of a long evolutionary process. Since 1965, further innovations have resulted in a plenitude of implant sizes, advanced surfaces, abutment implant connections, and abutment types [26].

While this wealth of resources has helped to circumvent many potential functional and esthetic complications, bone availability remains a crucial consideration for implant candidates; implants must be placed at sites with adequate hard and soft tissue [13,15,16,17]. This requirement, particularly in the vertical dimension, can be challenging to fulfill when an implant is planned at the site of an existing compromised tooth. In order to manage residual defects after extractions, especially of periodontally hopeless teeth, and place implants that maintain harmony with adjacent teeth, several techniques have been employed, including socket preservation, ridge augmentation, and forced eruption [15,16,17]. Unlike the former two, forced eruption, first documented for this purpose in 1993 by the Salama brothers, capitalizes upon the tooth’s ownership of its attachment apparatus to improve soft and hard tissue architecture to better receive a future implant restoration; tension of the periodontal ligament caused by orthodontic extrusion stimulates bone deposition at the alveolar crest [13,25,27,28].

#### 2.2.2. Advantages and Disadvantages

Implant location is a major factor in long-term success [15,26,29]. However, hard and soft tissue deficiencies are frequently encountered at planned implant sites, often resulting in compromised implant position [15,26,30,31]. Compared to ridge augmentation and other surgical procedures to manage the tissue around implant, forced eruption is relatively conservative and non-invasive and exemplifies the most natural phenomenon: the innate relationship between a tooth and its attachment apparatus [16]. Moreover, it limits the involvement of adjacent teeth and, by predictably increasing the soft and hard tissue volume, improves the esthetics, emergence profile, and crown-to-implant ratio of the future implant restoration [8,16]. As a result, additional grafting or soft tissue management surgeries secondary to implant placement may be avoided. The technique creates a leveled implant recipient site in harmony with the adjacent natural teeth [13,15,16,17,25,27,28].

However, caution must be exercised during vertical tooth movement; the elimination of occlusal interferences involves considerable tooth reduction, which can lead to sensitivity or even pulp exposure. Despite the fact that the tooth is to be extracted, prophylactic endodontic therapy may be necessary, thereby increasing cost [15,16]. The protracted treatment time is another disadvantage that may dissuade patients seeking swift results [13,15,17,32] (Table 3).

#### 2.2.3. Indications and Contraindications

With appropriate case selection, orthodontic extrusion serves as a useful strategy in developing implant sites (Table 4). General indications for orthodontic extrusion for implant site development include periodontally compromised or non-restorable teeth that are planned for extraction due to severe attachment loss, diagonal or horizontal fractures, large carious lesions, perforations, and root resorption, among other reasons [15,16]. In these instances, forced eruption of these teeth may induce the formation of supporting bone as well as enhancement of soft tissue [13,15,16,17,25,27,28]. However, teeth with a severely compromised attachment apparatus need to be evaluated carefully. The absence of the healthy attachment apparatus will result in an unfavorable outcome.

Since the attachment apparatus is required for bone and soft tissue remodeling during orthodontic tooth movement, absence of a healthy attachment apparatus, i.e., inflammation, is an absolute contraindication. In active periodontitis, extrusion of the tooth will result in coronal relocation of the tooth only, resulting in clinical attachment loss [15,16]. Other contraindications include ankylosis, hypercementosis, root proximity, vertical root fracture, and chronic inflammatory lesions. Such inflammatory lesions would lend unfavorable outcomes due to compromised tissue remodeling [13,15,16,17,32].

**Figure 2:**
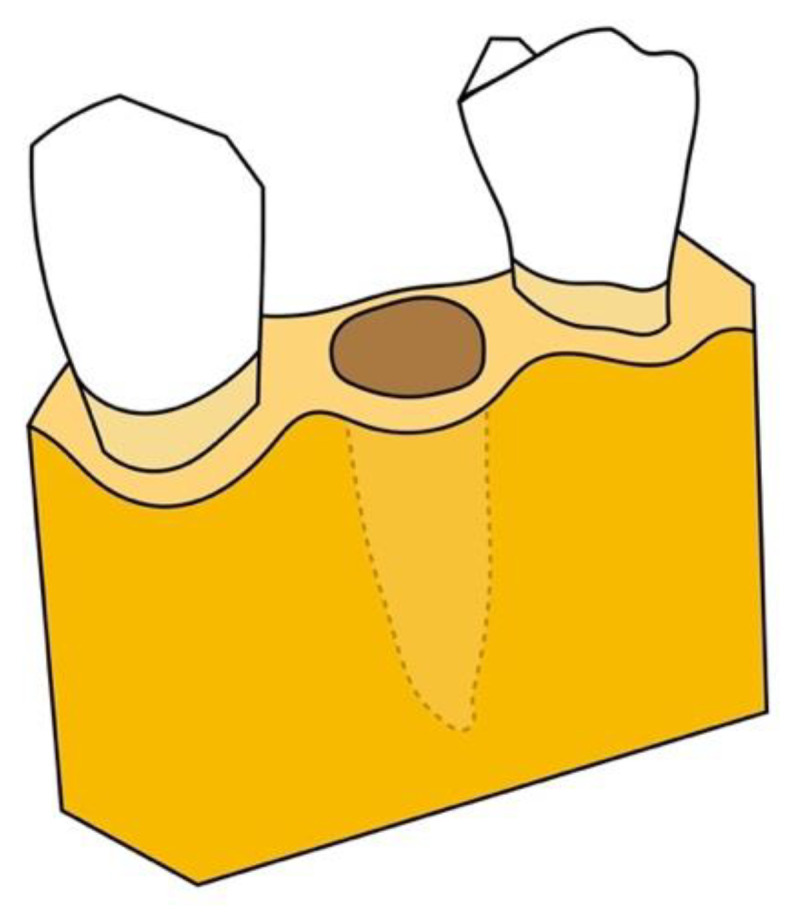
Type 1 defect: incipient defect environment has adequate regenerative and esthetic potential.

**Figure 3:**
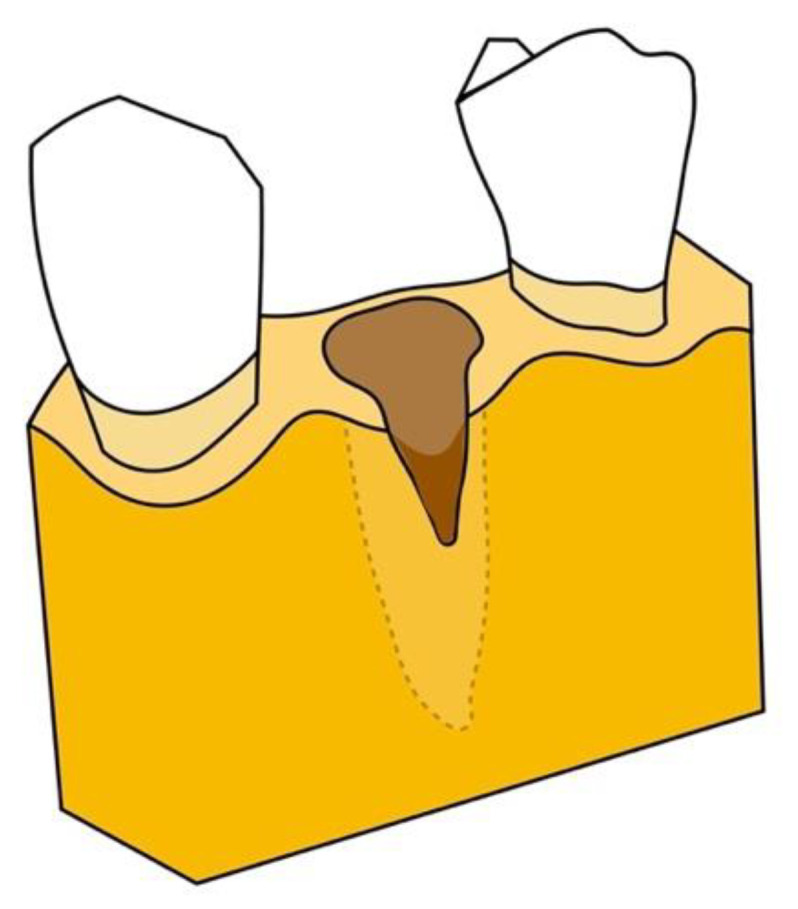
Type 2 defect: moderate bony defects may exhibit resorption of the buccal bone, dehiscence, and recession extending to the middle third of the root.

**Figure 4:**
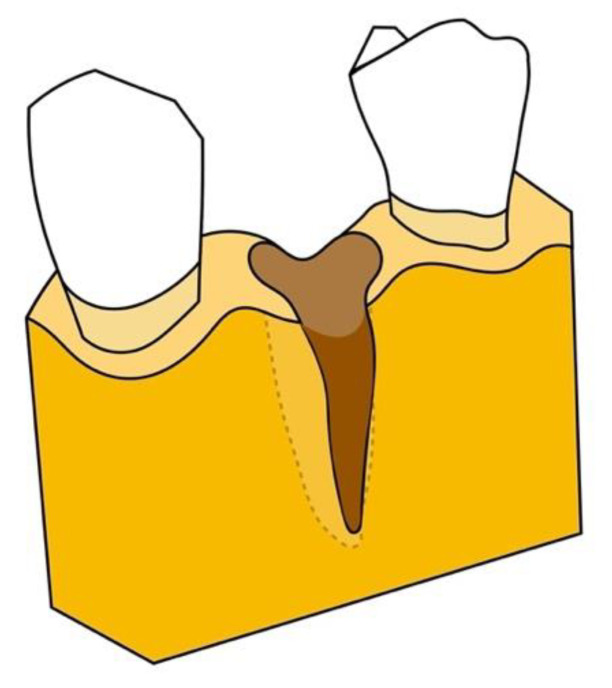
Type 3 defect: severe compromised defect with pronounced vertical and buccolingual osseous inadequacies, loss of the labial plate, and circumferential and angular defects.

According to Salama’s 1993 classification, extraction sites can be classified as Type 1, Type 2, or Type 3 extraction sites, based upon residual defect morphology and regenerative potential (Figure 2, Figure 3 and Figure 4). A Type 1 site is an incipient defect environment and has adequate regenerative and esthetic potential; these sites are highly amenable to forced eruption and immediate implant placement. The residual bone morphology and, concurrently, the regenerative and esthetic environment in a Type 2 site are more compromised; these moderate bony defects may exhibit resorption of the buccal bone, dehiscence, and recession extending to the middle third of the root. Orthodontic extrusion can be utilized to modify the Type 2 site and transform it into a Type 1 site. Therefore, both Type 1 and Type 2 sites are indicated for this procedure [13,15,16,17].

In contrast, the residual bone morphology in a Type 3 site is severely compromised with pronounced vertical and buccolingual osseous inadequacies, loss of the labial plate, and circumferential and angular defects. Hopeless teeth in these sites lack adequate surrounding periodontal apparatus for effective extrusion, which may lead to complications such as gingival recession and bone dehiscence. Indeed, such significant deformities are indications for guided tissue regeneration; forced eruption is contraindicated [13,15,16,17].

## 3. Biology of Forced Eruption

In general, orthodontic movement is composed microscopically of two zones: the pressure zone, located in the direction of tooth movement, and the tension zone, located opposite the direction of movement. The pressure zone is characterized by compression of the periodontal ligament (PDL) and disturbance of blood flow, leading to cell death through hyalinization. Subsequently, the hyalinized tissue and undermining bone are resorbed by macrophages and osteoclasts, respectively; this allows for tooth movement into the area of the newly resorbed bone [15,27,33,34]. Keeping end force levels constant, an initially light and gradually increasing force produces less hyalinization than a greater initial force [15]. Accordingly, a light orthodontic extrusive force is recommended. In contrast, the tension zone is characterized by stretching of the PDL and activation of blood flow, leading to promotion of osteoblast activity and osteoid formation. As teeth migrate coronally, the tension zone begets bone deposition in the area from which the tooth has moved [27,33,34]. In orthodontic extrusion, the tension created in the PDL by the extrusive force on the tooth leads to bone deposition and coronal migration of the attachment apparatus [16,34].

Considering the principles of orthodontic extrusion in developing more prosthetically and biomechanically suitable implant recipient sites, many “hopeless teeth” have a unique advantage prior to extraction that can enhance the ultimate functional and esthetic results. Indeed, the remaining attachment apparatus provides all three of the necessary components for successful regeneration; the socket acts as a natural scaffold, and the PDL provides a source of cells and signals [16].

However, the goal of forced eruption for establishment of ferrule effect is quite dissimilar. Since the desired outcome is crown lengthening, it is expected that the bone and soft tissue do not follow in the tooth’s coronal migration. To prevent this organic phenomenon, fiberotomy is performed to limit the formation of osteoid. This procedure, when performed regularly throughout the course of extrusion, may eliminate the need for additional crown lengthening surgery [12,20,22].

Hochman et al. classified soft tissue responses to orthodontic extrusion based on three pre-treatment parameters: sulcus or pocket depth, position of the mucogingival junction (MGJ) relative to the alveolar crest, and bone sounding or transgingival probing under local anesthesia (Table 5). The latter is performed to determine whether the attached gingiva is connected only to the root surface or also to the periosteum. In the Type 1 classification, the attached gingiva is connected to both the root and the bone, and extrusion will result in an increased width of attached gingiva. If the bone sounding measurement is greater than the keratinized tissue, then there is no connection between the attached gingiva and the bone; this is the case in the Type 2 classification, where the MGJ and thus all of the attached gingivae are connected only to the root surface. Orthodontic extrusion would still produce an increase of the soft tissue width, but the mucogingival junction would migrate with the tooth and the width of attached gingivae would remain unchanged. If the width of pre-treatment keratinized tissue was insufficient in a Type 2 case, additional soft tissue augmentation procedures may still be indicated prior to implant placement. The Type 3 classification is characterized by the presence of a periodontal pocket, where neither the MGJ nor the attached gingiva is connected to the root. There is no change in the width of attached gingivae or the position of the gingival margin until the pocket is everted [35].

## 4. Methodology and Efficacy

In order to achieve predictable and desirable outcomes, proper case selection is paramount. Considerations include crown-to-root ratio, root form, accessibility, smile line, and the condition of both the tooth to be erupted and the adjacent teeth. Since esthetics will be more difficult to achieve after eruption of narrow roots, wider roots are preferred [36]. Moreover, a tooth which has fractured 3 mm below the alveolar rest would have limited accessibility for placement of appliances for forced eruption, and would also not be a good candidate [21]. Patients with a high smile line, demonstrating more than just interproximal gingiva, would benefit from forced eruption for esthetic reasons as the gingival architecture around the tooth could be better preserved [7]. If adjacent teeth are more heavily restored or also compromised, perhaps the patient would benefit more from fixed or removable prosthodontics instead; younger patients with virgin adjacent teeth would be more favorable candidates due to their better regenerative potential [37].

**Figure 5:**
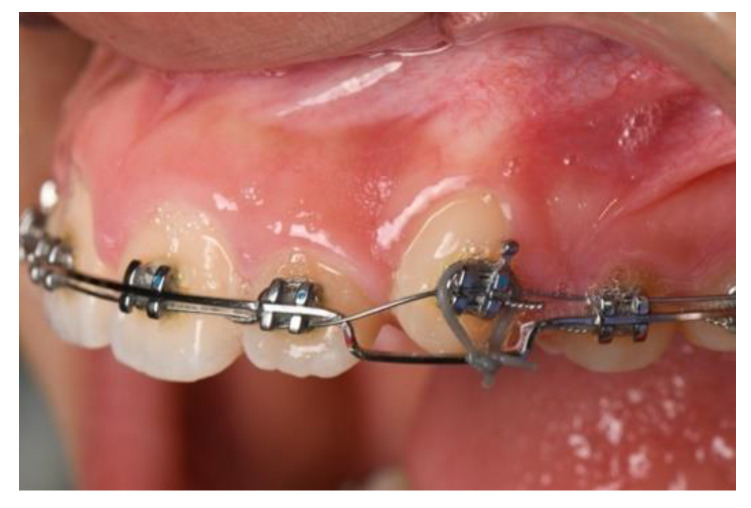
Fixed appliance used for forced eruption.

**Figure 6:**
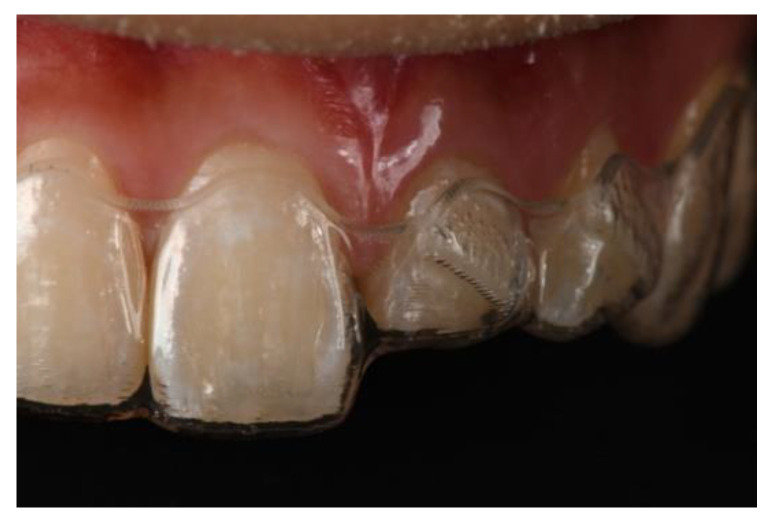
Removable appliance used for forced eruption.

There are two main methods to complete forced eruption: fixed appliances, which was first proposed by Ingber (Figure 5), and removable appliances (Figure 6), such as clear aligners.

The advantage of a fixed appliance is that it provides the clinician with full control of the movement, eliminating the variability of patient compliance and creating a more predictable treatment period. In fixed appliances, a ligature can be tied to the bracket slot, around the wings of a bracket, or a wire can be bonded to the tooth with composite. Forced eruption can be achieved by a sundry of techniques. Simon et al. used round wires bonded to the adjacent teeth and a hook placed in the canal of an endodontically treated tooth to achieve the extrusion. Elastics were attached to the hook and changed regularly to achieve the desired eruption. Nappen and Kohlan modified the technique by placing brackets on both the tooth to be extruded as well as the adjacent teeth [38]. Nickel titanium wires are attached to the brackets, and the tooth is extruded as the wire straightens. Other techniques to achieve forced eruption include utilization of mini-implant screws and magnets. Generally, a slower activation period with a rectangular wire has proven to yield the most predictable outcomes; this minimizes functional and esthetic complications such as buccal dehiscence, increased root prominence, or recession due to loss of torque control.

In cases where removable appliances are used, patient compliance is a major factor that may protract treatment time. The patient changes the aligner every week to allow for tooth movement, and the last aligner can be used as a retainer for retention. Placing attachments on both the buccal and lingual surfaces of the extruded tooth may be more effective for controlling the tooth movement through clear aligners. For both fixed and removable appliances, periodic occlusal adjustments are required to create space for eruption without causing traumatic interferences.

Generally, the recommended extrusion rate is 1–2 mm per month, and the accepted forces for anterior teeth and posterior teeth are 15 g and 50 g, respectively [17,39]. The applied force can be controlled by its type, magnitude, and duration. The eruptive phase in this technique usually requires four to six weeks, depending upon the amount of extrusion desired. Forced eruption has exhibited promising results in both gaining clinical crown length and developing implant sites in animal studies and clinical trials. Berglundh et al. (1991) demonstrated an average extrusion of 4.5 mm in a dog model in 16 weeks [40]. The probing depths remained unchanged and the gingival margins receded only 0.5 mm [40]. Their observation agrees with those of Batenhorst et al. (1974) and Simon et al. (1980), which likewise showed that vertical extrusion of teeth can be accomplished without inflicting major undue damage to the periodontal attachment apparatus [33,41]. Ingber extruded non-restorable teeth by 3 to 5 mm, and subsequently performed periodontal surgery to level the osseous crests [11]. As summarized by a recent systematic review of forced eruption for implant site development by Somar et al., an average of 0.5–1 mm of active extrusion per month was achieved by Chou et al. and Amato et al. using either a partial or full orthodontic bracket setup [39,42,43]. Keceli et al. also reported successful treatment with an extrusion rate of 1 mm per week and three weeks of stabilization [44]. In these studies, the efficacy of bone augmentation ranged from 69% to 100%. Amato et al. reported an average of 65% keratinized soft tissue augmentation [39].

Even though the forced eruption is able to modify the periodontal apparatus, orthodontic tooth movement can relapse if proper retention or stabilization has not been achieved. The ideal timeline for stabilization before restoration is not clear in the literature, but enough time should be allowed for soft tissue healing; stabilization periods of between one and six months have been reported [15,27]. Typically, sites undergoing orthodontic extrusion for prosthetic purposes require less treatment time than those for implant site development, as additional time is required to allow maturation of the alveolus so that adequate stability can be expected with immediate implant placement [16,17].

However, evaluation of treatment period involves consideration of not only the extrusion period, but also the need for any additional surgical procedures after extrusion to complete the case. When the goal is to increase clinical crown length, fiberotomy is usually performed to aid in orthodontic movement [12,20,22]. As discussed by Pontoriero, the fiberotomy involves severing the connective tissue attachments around the tooth [12]. When performed on a weekly basis, the coronal migration of the periodontal attachment apparatus can be successfully decreased, albeit not altogether prevented, as the tooth is extruded [40]. Weekly fiberotomy can minimize but perhaps not completely prevent concurrent coronal migration of the attachment apparatus. Berglundh demonstrated an average of only 1.6 mm of gingival margin recession when teeth were extruded 4.3 mm in a dog model [40]. However, Pontoriero (1987), Kozlovsky (1988), and Carvalho (2006) reported nearly no changes in soft tissue positioning [12,20,22]. Further studies are necessary to truly elucidate the efficacy of fiberotomy in combination with extrusion in preventing the need for additional gingival or osseous corrections. Should additional crown lengthening surgery be required after extrusion, the cost and duration of treatment would certainly increase.

## 5. Conclusions

Based on our review of the available literature, orthodontic extrusion is a valuable treatment option in the management of compromised or non-restorable teeth. Unlike traditional crown lengthening surgery, forced eruption combined with fiberotomy can convert a tooth deemed non-restorable due to subgingival caries or perforations violating the biological width into a restorable tooth with adequate ferrule without compromising the periodontium of the adjacent teeth or impairing the crown-root ratio of the final restoration. Especially in the esthetic zone, this technique can better preserve the pre-procedural gingival architecture and avoid recession and sensitivity in the treatment area. Even if a tooth is deemed hopeless due to severe attachment loss, its natural relationship with its attachment apparatus still presents an inimitable asset that can only be unlocked through orthodontic extrusion. Indeed, forced eruption may serve as the most physiologic and noninvasive approach to establishing a favorable soft and hard tissue environment for implant placement. Nevertheless, interdisciplinary planning and meticulous case selection are essential to ensuring treatment success. There is a paucity of controlled studies that have investigated the long-term stability of orthodontically extruded sites or the efficacy of fiberotomy in obviating the need for further crown lengthening surgeries, and further examination of these areas is required. Moreover, there has not yet been a systematic review that can provide clinicians with clear, evidence-based guidelines regarding clinical indications, management, and possible complications of forced eruption. It is our intention to further investigate this topic in order to provide a higher level of evidence-based literature to guide future clinical practice.

## Figures and Tables

**Table 1:** Advantages and Disadvantages of Forced Eruption for Crown Lengthening.

Advantages	Disadvantages
Non-invasive	Considerable occlusal reduction
Limits involvement of adjacent teeth	Endodontic therapy may be required
Preserves supporting bone	Prolonged treatment time
Improves esthetics	Minor periodontal surgery may still be indicated
Maintains crown-to-root ratio	Additional cost

**Table 2:** Indications and Contraindications of Forced Eruption for Crown Lengthening.

Indications	Contraindications
Subgingival or intraosseous caries	Active periodontal inflammation
Isolated periodontal defects	Ankylosis
Horizontal fractures	Hypercementosis
Perforations	Root proximity
Internal and external root resorptions	Vertical root fracture
	Chronic inflammatory lesions
	Risk of furcation exposure
	Poor crown-to-root ratio
	Insufficient prosthetic space

**Table 3:** Advantages and Disadvantages of Forced Eruption for implant site development.

Advantages	Disadvantages
Non-invasive	Considerable occlusal reduction
Limits involvement of adjacent teeth	Endodontic therapy may be required
Increases soft and hard tissue volume	Prolonged treatment time
Improves esthetics and future emergence profile	Additional cost
Improves future crown-to-implant ratio	
May obviate additional grafting surgeries	

**Table 4:** Indications and contraindications of forced eruption for implant site development.

Indications	Contraindications
Non-restorable teeth	Active periodontal inflammation
Large carious lesions	Ankylosis
Internal and external root resorptions	Hypercementosis
Perforations	Root proximity
Diagonal/horizontal fractures	Vertical root fracture
Type 1 and Type 2 extraction sites	Chronic inflammatory lesions
	Type 3 extraction sites

**Table 5:** Hochman’s Classification of soft tissue responses to orthodontic extrusion.

	Attached Gingiva Connection	Width of Attached Gingiva	Position of MGJ	Width of Soft Tissue
Type 1	Root and bone	Increases	No change	Increases
Type 2	Root only	No change	Coronal	Increases
Type 3	Neither	No change	No change	No change

## Data Availability

Data sharing not applicable.
